# Sublethal concentrations of clothianidin affect honey bee colony growth and hive CO_2_ concentration

**DOI:** 10.1038/s41598-021-83958-8

**Published:** 2021-02-23

**Authors:** William G. Meikle, John J. Adamczyk, Milagra Weiss, Janie Ross, Chris Werle, Eli Beren

**Affiliations:** 1 Carl Hayden Bee Research Center, USDA-ARS, 2000 E. Allen Rd, Tucson, AZ 85719 USA; 2 Thad Cochran Southern Horticultural Laboratory, USDA-ARS, P.O. Box 287, Poplarville, MS 39470 USA

**Keywords:** Environmental impact, Agroecology

## Abstract

The effects of agricultural pesticide exposure upon honey bee colonies is of increasing interest to beekeepers and researchers, and the impact of neonicotinoid pesticides in particular has come under intense scrutiny. To explore potential colony-level effects of a neonicotinoid pesticide at field-relevant concentrations, honey bee colonies were fed 5- and 20-ppb concentrations of clothianidin in sugar syrup while control colonies were fed unadulterated syrup. Two experiments were conducted in successive years at the same site in southern Arizona, and one in the high rainfall environment of Mississippi. Across all three experiments, adult bee masses were about 21% lower among colonies fed 20-ppb clothianidin than the untreated control group, but no effects of treatment on brood production were observed. Average daily hive weight losses per day in the 5-ppb clothianidin colonies were about 39% lower post-treatment than in the 20-ppb clothianidin colonies, indicating lower consumption and/or better foraging, but the dry weights of newly-emerged adult bees were on average 6–7% lower in the 5-ppb group compared to the other groups, suggesting a nutritional problem in the 5-ppb group. Internal hive CO_2_ concentration was higher on average in colonies fed 20-ppb clothianidin, which could have resulted from greater CO_2_ production and/or reduced ventilating activity. Hive temperature average and daily variability were not affected by clothianidin exposure but did differ significantly among trials. Clothianidin was found to be, like imidacloprid, highly stable in honey in the hive environment over several months.

## Introduction

Neonicotinoid pesticides have been suspected as a major factor in the decline in pollinator abundance, and a global survey of honey samples has shown that the exposure of honey bees to neonicotinoid pesticides is cosmopolitan^1^. Among neonicotinoid pesticides, thiamethoxam and its metabolite, clothianidin, are among the most widely-used and pose the greatest risk for honey bees (*Apis mellifera* L.)^2,3^. Clothianidin exposure has been found to affect honey bee grooming, hygienic behavior and neural gene expression^4–6^; memory processing^7^; drone semen quality^8^; and has been associated with increased P450 gene expression^9^ indicating active detoxification. Clothianidin has been found in some studies to increase worker mortality^10–12^ and when combined with λ-cyhalothrin was shown to affect adult bee weight^9^. Exposure of honey bees to clothianidin and nutritional stress synergistically reduced bee survival and haemolymph sugar levels^13^. Honey bees exposed to neonicotinoids have been found to have higher *Varroa* and *Nosema* densities^14–17^ and reduced social immunity^11^. Imidacloprid, perhaps the most heavily used neonicotinoid, has been shown to affect brood production, queen replacement, foraging activity and winter survivorship when applied at sublethal concentrations in pollen diet^14^. When applied at a 5-ppb concentration in sugar syrup, imidacloprid has been found to affect honey bee colony thermoregulation and adult bee maturation^18–20^, and has significantly affected bumble bee colony behavior and thermoregulation^21^.

Sublethal pesticide exposure may affect aspects of honey bee ecology and social organization, but in the case of clothianidin, observations of negative impacts in managed manipulative field studies have not been consistent. Different research groups have reported no effects of field-realistic concentrations of clothianidin on colony-level growth or behavior^22,23^, or on colony winter survival^24^. A large-scale study in Germany in which bee colonies were allowed to forage on oilseed crops treated with clothianidin found no effects on development of colony strength, brood success, honey yield or levels of pathogen infection^25^. Similarly, a field study involving “mini-colonies” challenged with both *Nosema* and clothianidin found no effect of clothianidin treatment on mortality or flight activity, and while the lifespans of *Nosema* infected bees were reduced compared to non-infected bees a combination of pesticide and pathogen did not reveal any synergistic effect^26^. Experiments with imidacloprid have also had mixed results with respect to colony growth and thermoregulation^19,20,27^.

One way to measure the impact of stressors such as pesticides on bee colonies is through the use of sensors, here defined as devices that provide continuous data on physical parameters such as weight and temperature. For example, continuous hive weight data show daily hive weight gain due to foraging, as well as changes in colony resources due to food consumption, robbing or reproductive swarming^28,29^. Continuous hive temperature data show colony thermoregulation behavior due to the presence of brood, which require a temperature of 34–36 °C^30,31^, and to low ambient temperatures^28,32^. Colony thermoregulation can be affected by subspecies^33^, within-colony genetic diversity^34^, phenological status^35^ and pesticide exposure^20,21,36^. Hive CO_2_ concentrations are also controlled by honey bee colonies within the hive and exhibit strong changes on a daily basis^37,38^. When concentrations of CO_2_, O_2_ and N_2_ within a hive were manipulated, only changes in CO_2_ provoked fanning behavior within the colony^39^. By controlling CO_2_ concentration in bee hives, bees actively maintain a reversible hypoxia and a reduced metabolic rate that, researchers hypothesized, allows them to conserve water and energy, as well as increase activity on short notice^40^. CO_2_ concentration is fundamentally different from measures such as temperature and humidity in that, because bees produce (and cannot absorb) CO_2_, and do not produce any other gases in sufficient quantity to displace appreciable amounts of CO_2_, hive CO_2_ concentrations are generally equal to or higher than concentrations outside the hive.

To test the hypothesis that low concentrations of clothianidin can have measurable effects on bee colony growth and behavior, honey bee colonies were fed with field-relevant concentrations (5 and 20-ppb) of clothianidin in sugar syrup over approximately six weeks. The treatment was applied in in-hive feeders to simulate pesticide exposure via nectar collection in the field. Exposure of the individual bees in a given colony would vary- bee colonies typically have larvae and adults of different ages so some bees would only be exposed as adults, some only as larvae, and some for their entire lives. Pesticide levels would be elevated in the stored honey so essentially all the bees in treated colonies would have had some degree of exposure by the end of the experiment. Treatment concentrations were based both on those used in previous studies on the exposure of bees to neonicotinoids^20,27^ and on values reported from large-scale surveys^41^. Acute oral LD_50_ of clothianidin to honey bees is about 4 ng per bee (Pesticide Properties Database: https://sitem.herts.ac.uk/aeru/ppdb/en/atoz_insect.htm), or a single dose of about 200 mg of the 20-ppb syrup, which exceeds the maximum estimated daily intake for even high-consumption nectar foragers^42^.

Colony size, growth and behavior were monitored for several months post-treatment using sensors and hive inspections, and avoiding methods that rely strictly on visual estimation. Colony size in terms of adult bee mass and area of sealed brood (late instar larvae and pupae) was measured using a weight difference method and a photograph analysis program^43^. Colony growth was estimated from the daily hive weight change, and colony behavior was observed by monitoring internal hive temperatures (i.e. thermoregulation), which have detected sublethal pesticide effects in other studies^20,28^, and internal CO_2_ concentrations. Weather and landscape have been found to play important roles in toxicological studies on bee colonies^44,45^, so the experiment was conducted twice (2017 and 2018) at a site in Arizona. A third experiment was conducted in Mississippi to determine whether effects observed in Arizona were consistent in a very different environment. Some measurements were made only at the Arizona site, including newly-emerged bee dry weight, hive CO_2_ concentration, and pesticide residue concentrations in honey and wax.

## Results

Two studies were conducted at the Santa Rita Experimental Range south of Tucson, AZ (31° 47′ 1.51ʺ N, 110°51′ 37.39ʺ W). The first study ran from May 2017 to March 2018 (hereafter AZ 2017) and the second study, from May 2018 to February 2019 (hereafter AZ 2018). The apiary was surrounded by unmanaged native plants, e.g., mesquite (*Prosopis* spp.), cactus (mostly *Opuntia* spp.), creosote (*Larrea* spp.) and wildflowers. No commercial agriculture existed within 10 km of the apiary. The site had 286 mm of precipitation during AZ 2017 and 540 mm during AZ 2018. Bee colonies were provided with a constant water source during both experiments, but low rainfall inhibited flowering, reducing the nectar and pollen forage and colonies in AZ 2017 required supplemental feeding in November. An additional study was conducted in Poplarville, MS (30° 50′ 2.59ʺ N, 89° 32′ 52.45ʺ W) from May 2018 to March 2019 (hereafter MS 2018). The Mississippi site received 1395 mm rainfall during MS 2018. An overview of the response variables and data for all experiments is provided (Table 1).

**Table 1:** Overview of experimental design, colony survivorship and response variables. *NEB*  newly emerged bee. “Start” was defined as the beginning of the treatment period in mid-July; “End” was defined as February 12 of the following year; “No. assess.” means the number of colony assessments.

Experiment	No. colonies								
	Start	End	No. assess	NEB mass	Hive weight	Hive temp	Hive [CO_2_]	Pesticide residues	*Varroa* levels
AZ 2017	18	12	5	Yes	Yes	Yes	No	Yes	Yes
AZ 2018	18	7	5	Yes	Yes	Yes	Yes	Yes	Yes
MS 2018	15	14	3	No	Yes	Yes	No	No	Yes

### Hive survivorship

**Figure 1:**
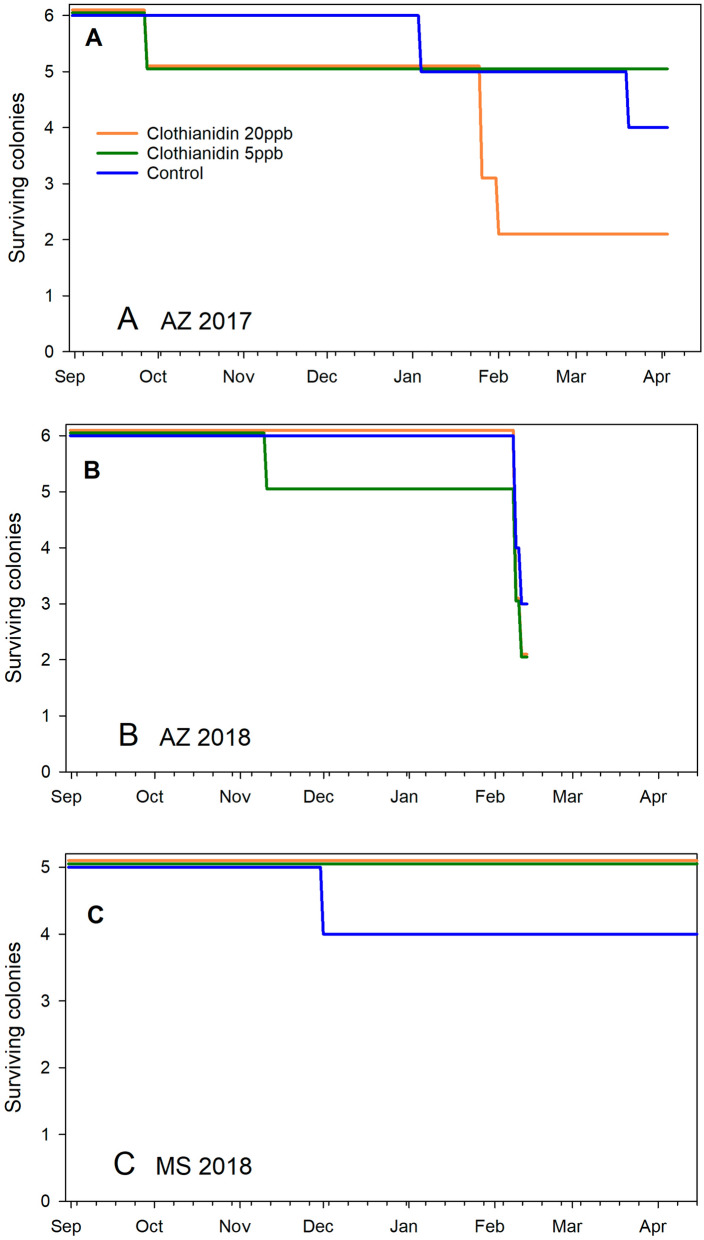
Honey bee colony survivorship from 1 Sept. to 15 April for each of the 3 treatment groups: Clothianidin 20-ppb (orange), clothianidin 5-ppb (green), control (blue) across 3 experiments. (**A**) AZ 2017; (**B**) AZ 2018; (**C**) MS 2018.

No significant differences in hive survivorship were observed among treatment groups (*p* = 0.55 for the 30th percentile, *p* = 0.38 for the 50^th^ percentile, and *p* = 0.48 for the difference between the 30th and 40th percentiles) (Fig. 1).

### Syrup consumption

The amount of syrup offered to the colonies each year was partly a function of the amount they consumed (that is, removed from the internal feeder). Colonies in AZ 2017 were offered a total of 30 kg syrup over 38 d and consumed on average 85 ± 3%, while colonies in AZ 2018 were offered 35 kg syrup over 39 d and consumed on average 97 ± 1%. A comparison of syrup consumption between the two Arizona experiments showed that colonies in AZ 2018 consumed significantly more than colonies did the previous year (*p* < 0.001) but neither treatment group nor their interaction were significant. Colonies in MS 2018 were fed a total of 36 kg syrup over 38 d and consumed it all.

### Adult bee mass

**Figure 2:**
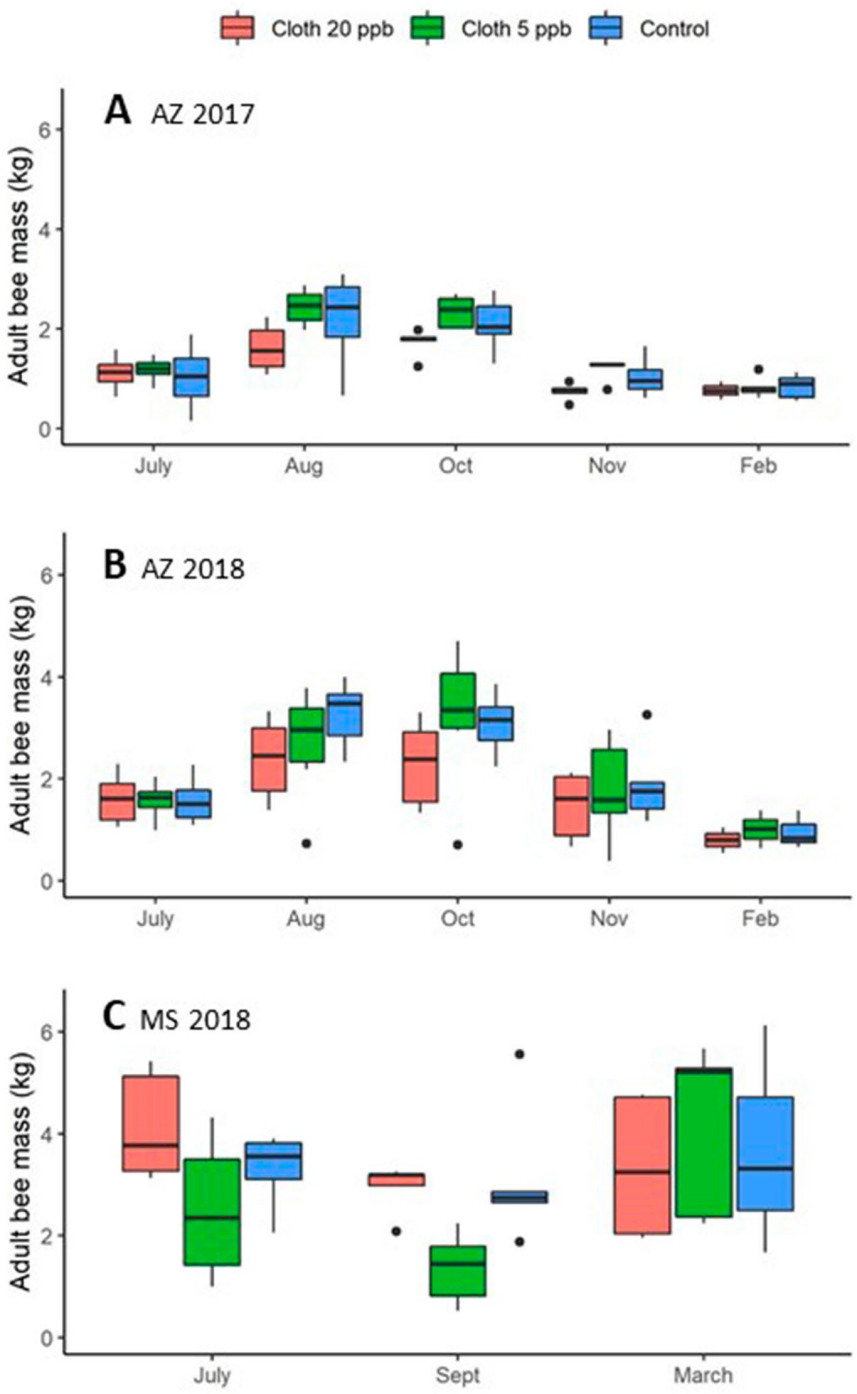
Average adult bee mass (kg) per colony for each of 3 treatment groups: Clothianidin 20-ppb (orange), clothianidin 5-ppb (green), control (blue). (**A**) AZ 2017; (**B**) AZ 2018; (**C**) MS 2018. Boxes are defined as 1.58 * IQR/n^0.5^, where IQR is the inter-quartile range and n is the number of data points.

Considering all three experiments, both treatment and experiment had significant effects on adult bee mass for the 1st sampling occasion after the end of treatment, while the interaction term was not significant (Fig. 2, Table 2, Supplementary Tables S1 and S2 online). Post hoc pairwise contrasts showed that colonies in the clothianidin 20-ppb treatment had significantly lower adult bee mass (2.18 ± 0.14 kg) at that point than colonies in the control treatment (average ± s.e.) (2.75 ± 0.17 kg). Pairwise contrasts did not detect any significant differences among experiments at the *p* = 0.05 level. Restricting the analysis to the Arizona experiments, treatment also had a significant effect across the 4 post-treatment hive evaluations (Supplementary Tables S3 and S4 online). However, post hoc pairwise contrasts did not reveal any significant differences at the *p* = 0.05 level among treatment groups, and the interaction between treatment and sampling occasion was not significant.

**Table 2:** Summary of results for MANOVA analyses for treatment and experiment on different response variables. “All” indicates that all three experiments were included in the analysis; “AZ only” indicates that only the Arizona experiments were included. The covariates in all analyses were pre-treatment values of those variables. Newly Emerged Bee (NEB) dry weight data were only collected in Arizona and CO_2_ data were only collected in the AZ 2018 study.

Response variable	Datasets	Sample periods (post treatment)	Treatment	Exp	Treat. x Exp
Adult mass (kg)	All	1st sample (Sept.)	**0.0240**	**0.0180**	0.1290
Adult mass (kg)	AZ only	Sept., Oct., Nov	**0.0456**	0.5053	0.5712
Brood area (cm^2^)	All	1st sample (Sept.)	0.5131	0.2729	0.7098
Brood area (cm^2^)	AZ only	Sept., Oct., Nov	0.5532	0.1197	0.7798
Food resources (kg)	All	1st sample (Sept.)	0.3724	**< 0.0001**	0.6879
Food resources (kg)	AZ only	Sept., Oct., Nov	0.4346	**< 0.0001**	0.2195
Daily hive wt. change (g/day)	All	1 Sept. – 31 Oct	0.8908	**< 0.0001**	**0.0324**
Daily hive wt. change (g/day)	AZ only	1 Sept. – 31 Oct	**0.0301**	**< 0.0001**	0.0759
Temperature average (°C)	All	1 Sept. – 31 Oct	0.4084	**< 0.0001**	0. 7020
Temperature amplitude (°C)	All	1 Sept. – 31 Oct	0.2159	**< 0. 0001**	0. 5664
NEB dry weight (g)	AZ only	Aug	**0.0413**	0.2485	0.1008
NEB dry weight (g)	AZ 2018 only	Aug., Oct	**0.0046**	–	–
Log CO_2_ average (ppm)	AZ 2018 only	1 Sept.– 31 Oct	**0.0003**	–	–
Log CO_2_ amplitude (ppm)	AZ 2018 only	1 Sept.– 31 Oct	**0.0041**	–	–
Log Varroa fall per day	AZ only	Aug. – Nov	0.4160	**< 0.0001**	0.3474

Numbers in bold indicate signficance at P < 0.05.

### Brood surface area

**Figure 3:**
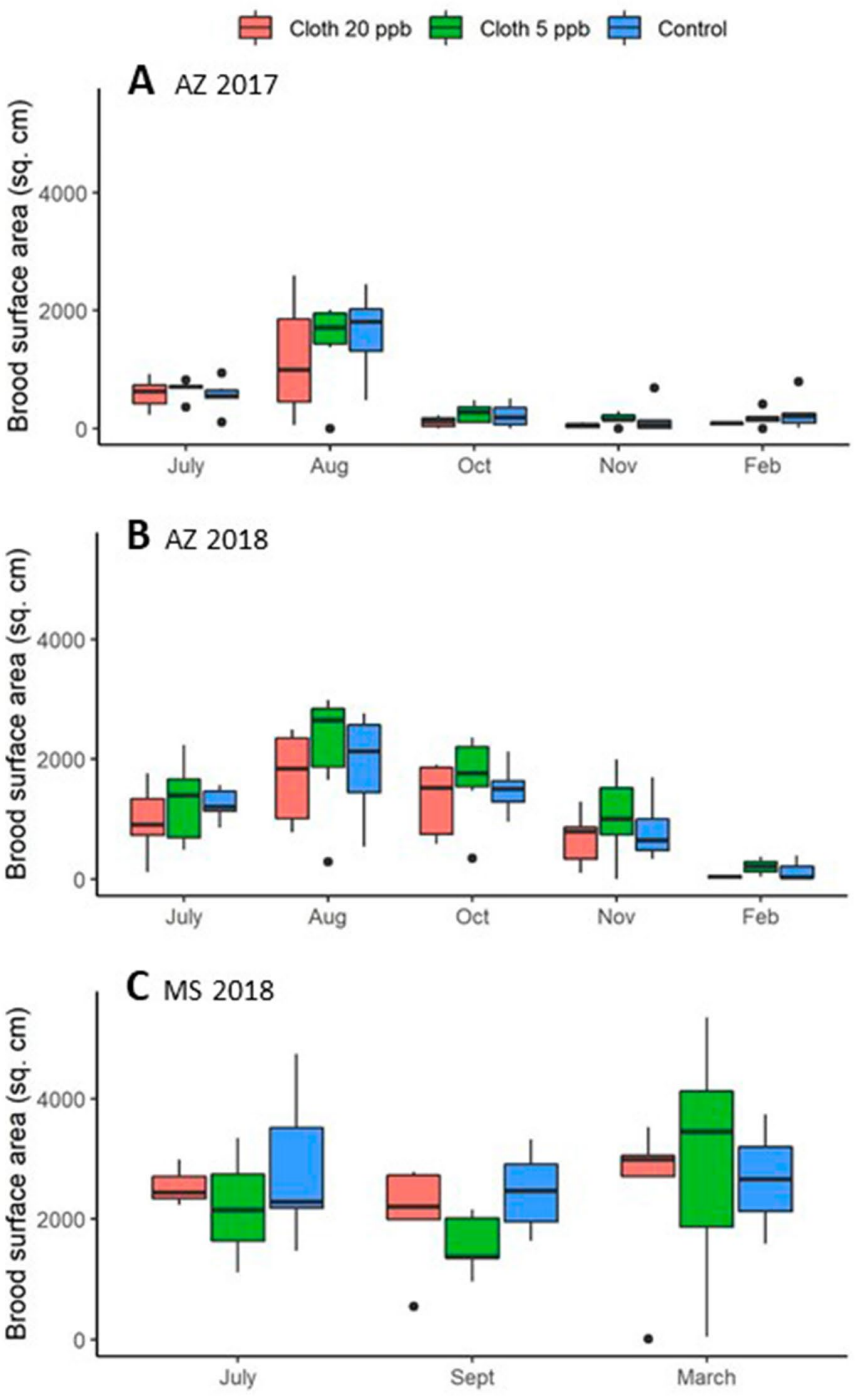
Average brood surface area (cm^2^) per colony for each of 3 treatment groups: Clothianidin 20-ppb (orange), clothianidin 5-ppb (green), control (blue). (**A**) AZ 2017; (**B**) AZ 2018; (**C**) MS 2018. Boxes are defined as 1.58 * IQR/n^0.5^, where IQR is the inter-quartile range and n is the number of data points.

Neither treatment nor experiment had significant effects on brood surface area, either considering all experiments on the 1st sampling occasion after the end of treatment, or with across the 4 post-treatment hive evaluations in Arizona (Fig. 3, Supplementary Tables S5 and S6 online). Brood production was very low by October in AZ 2017.

### Total food resources

The experiment factor had a significant effect on food resources, considering all experiments on the 1st sampling occasion after the end of treatment, but treatment did not (Supplementary Tables S7-S10 online). Pairwise post hoc contrasts showed that hives in the AZ 2017 experiment had significantly fewer resources on average, when taking pre-treatment resources into account, then hives in either of the other two experiments. Prior to treatment, hives in MS 2018 had 9.62 ± 0.99 kg food resources, hives in the AZ 2018 had 4.78 ± 0.41 kg and hives in AZ 2017 had on average of 4.06 ± 0.28 kg. Post-treatment, hives in MS 2018 had on average 21.85 ± 1.32 kg, hives in AZ 2018 had on average 16.36 ± 0.57 kg and hives in AZ 2017 had on average 10.24 ± 0.68 kg. Comparing the two Arizona experiments across the three sampling occasions in the fall (the February sampling occasion was omitted because of effects from the spring nectar flow), the experiment factor was again significant while treatment was not, and average resources per hive were significantly higher in AZ 2018 at all three sampling occasions.

### Newly emerged bee (NEB) dry weight

**Figure 4:**
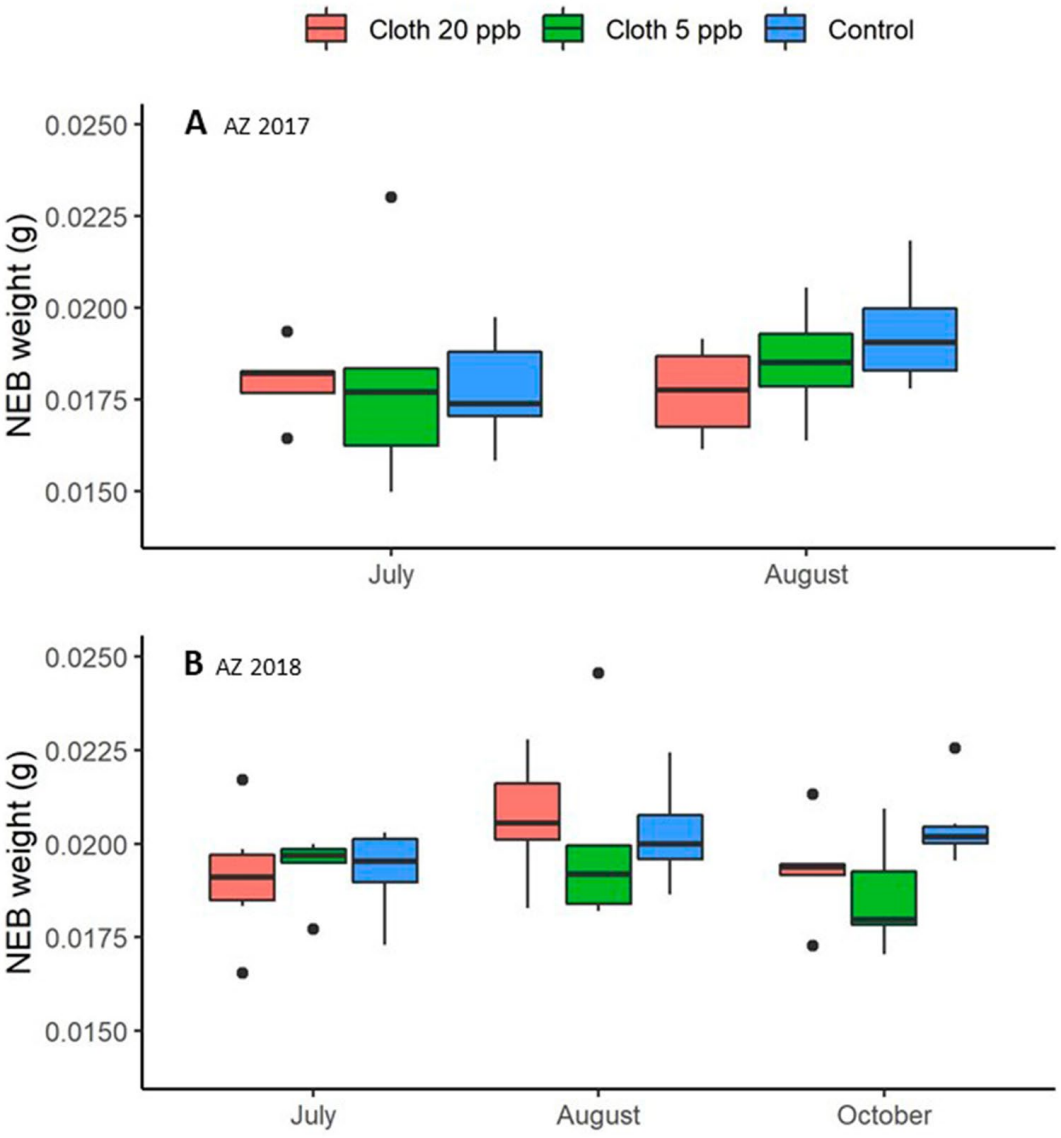
Average Newly Emerged Bee (NEB) dry weights for each of 3 treatment groups: Clothianidin 20-ppb (orange), clothianidin 5-ppb (green), control (blue). (**A**) AZ 2017 (2 sampling dates); (**B**) AZ 2018 (3 sampling dates). Boxes are defined as 1.58 * IQR/n^0.5^, where IQR is the inter-quartile range and n is the number of data points.

Considering AZ 2017 and AZ 2018 together for August (the only post-treatment sampling date included in both experiments), treatment was significant, while experiment and the treatment x experiment interaction were not (Supplementary Tables S11 and S12 online). Pairwise contrasts showed that the NEB dry weights from the clothianidin 5-ppb group were about 6.1% smaller, on average 0.0186 g, than NEBs from the control group, on average 0.0198 g (*p* = 0.0440). Data for AZ 2018 included an additional sampling occasion, so another analysis was conducted (Supplementary Tables S13 and S14 online). In that analysis, treatment had a significant effect on NEB dry weight for the August and October samples (Fig. 4) and neither sampling date nor the interaction term was significant. Post hoc contrasts also showed that average NEB dry weight from the clothianidin 5-ppb treatment group was about 7.2% smaller than that of the control group (0.0189 g vs. 0.0204 g) (*p* = 0.0054) and 5.2% smaller than the clothianidin 20-ppb group (0.0200 g) (*p* = 0.0310). The control and clothianidin 20-ppb groups were not significantly different.

### Daily hive weight change

**Figure 5:**
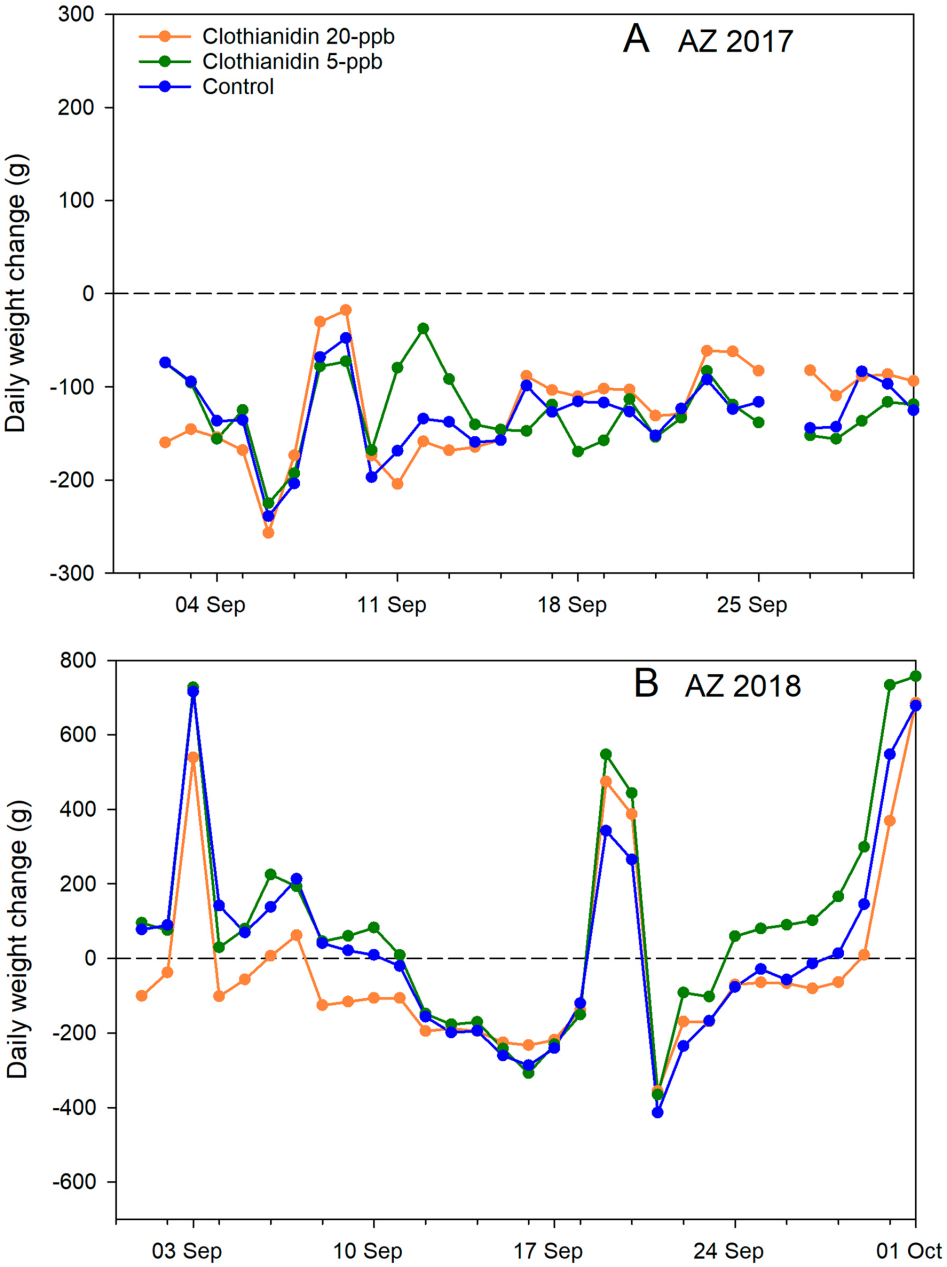
Average colony daily weight change (g) for each of 3 treatment groups: Clothianidin 20-ppb (orange), clothianidin 5-ppb (green), control (blue) for the month of September for 2 experiments. (**A**) AZ 2017; (**B**) AZ 2018.

Considering all experiments during the post-treatment period from 1 Sept. to 31 Oct. (the point at which colonies had largely ceased foraging), treatment did not have a significant effect (*p* = 0.89) but the experiment factor did (*p* < 0.0001) as did the interaction term (*p* = 0.03) (Supplementary Tables S15 and S16 online). Post hoc contrasts showed that colonies in AZ 2017 had significantly greater weight loss, 187 ± 16 g/day, than those in AZ 2018, 9 ± 7 g/day, while colonies in MS 2018 gained 6 ± 3 g/day. Considering the two Arizona experiments from 1 Sept. to 31 Oct., treatment was found to have a significant effect on daily weight change (*p* = 0.03) and the interaction of treatment and experiment was not significant, indicating the effect was consistent between the two years (Fig. 5) (Supplementary Tables S17 and S18 online). Post hoc contrasts showed that colonies fed 20-ppb clothianidin on average lost more weight per day post-treatment, on average 127 ± 20 g than did colonies fed 5-ppb clothianidin, 90 ± 19 g. Control colonies, which lost on average 95 ± 16 g per day, were not significantly different from either group. Colonies in AZ 2017 lost much more weight per day overall, on average 196 ± 19 g, than did colonies the following year, on average 13 ± 8 g per day. Daily hive weight change in the winter (1 Dec.–31 Jan.) was not affected by treatment (Supplementary Table S19 online).

### Hive temperature average and variability

**Figure 6:**
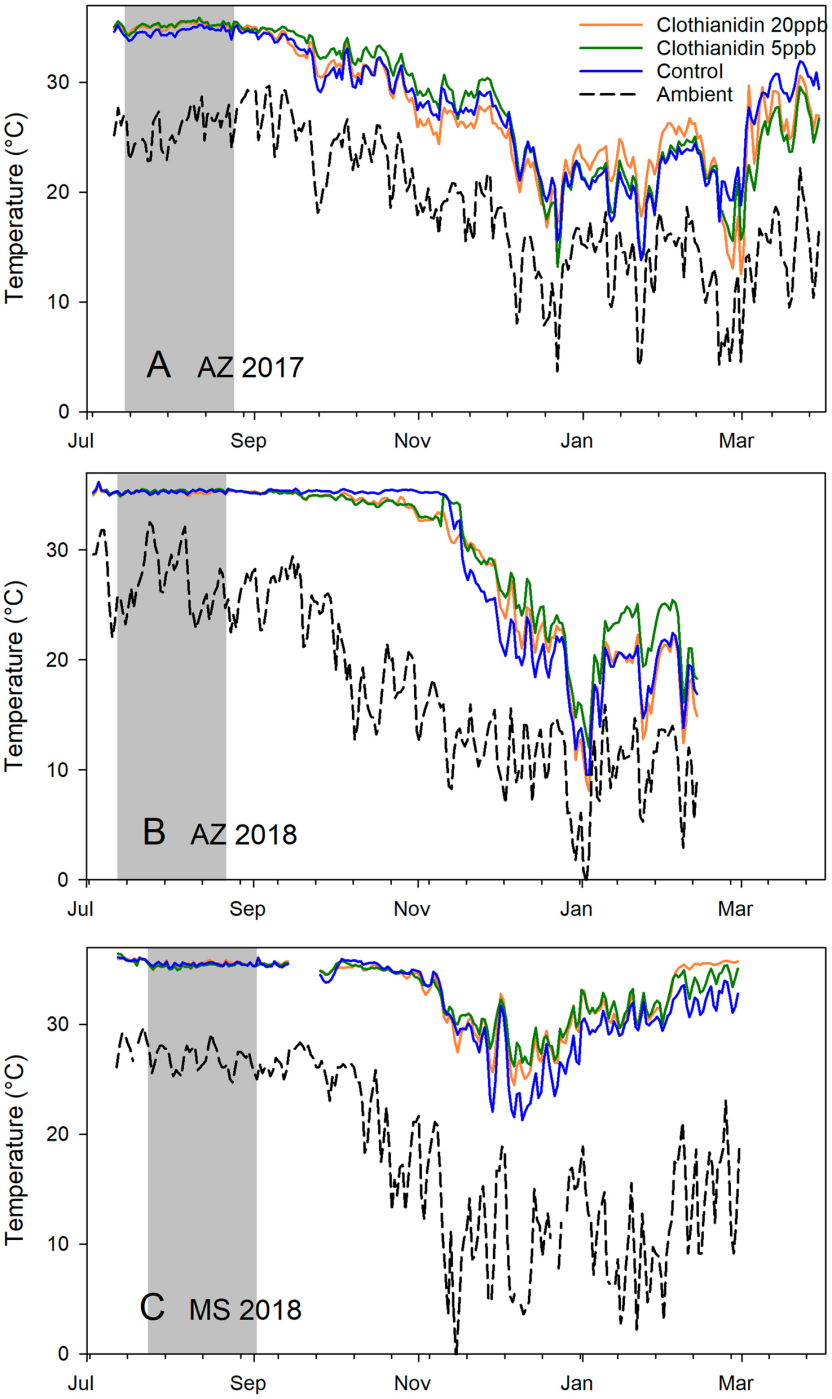
24 h running average internal hive temperature (°C) per hour for each of 3 treatment groups: Clothianidin 20-ppb (orange), clothianidin 5-ppb (green), control (blue) compared to ambient temperatures (black) across 3 experiments. The gray area shows the treatment period. (**A**) AZ 2017; (**B**) AZ 2018; (**C**) MS 2018.

**Figure 7:**
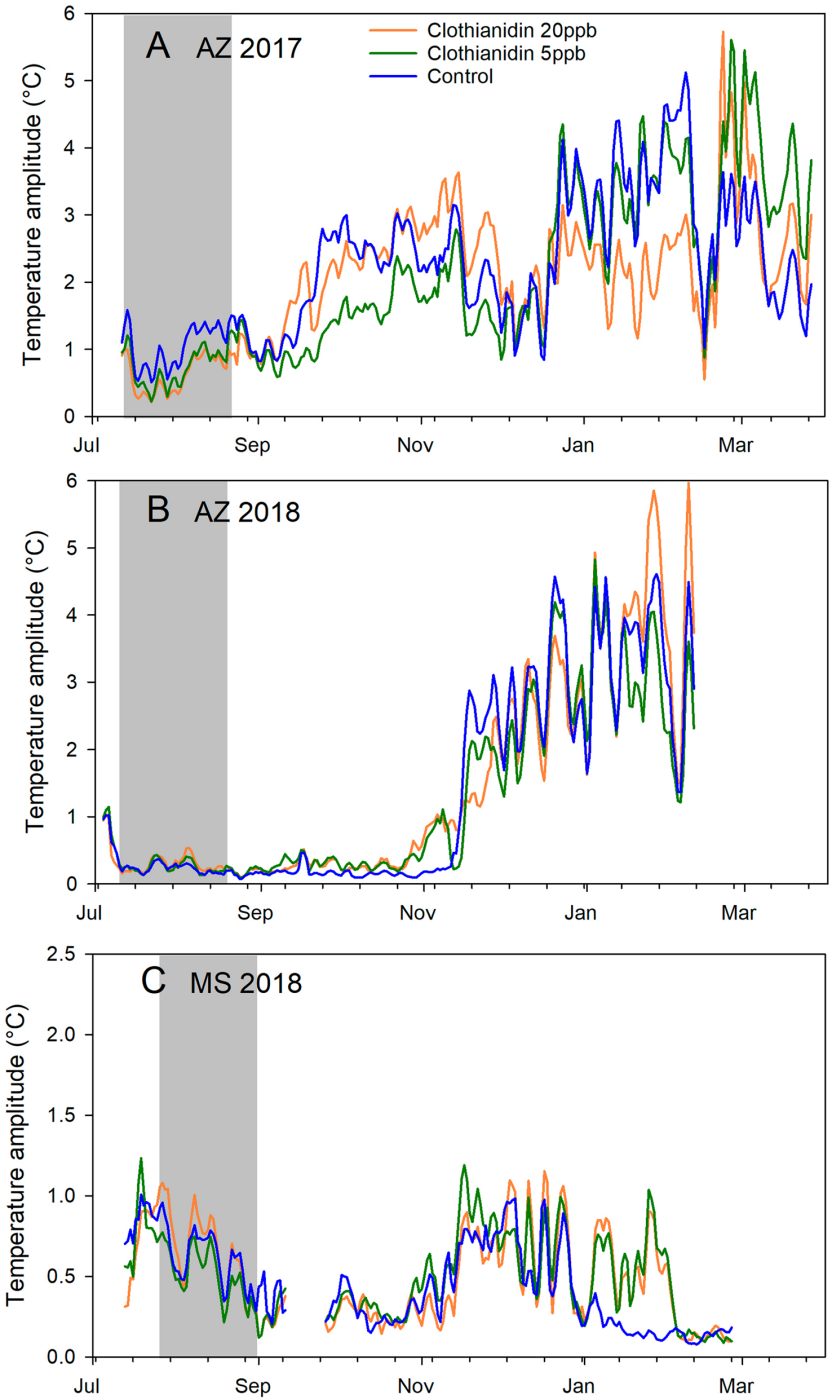
Average daily amplitudes of sine curves fit to within-day temperature changes per day (see text for details) for each of 3 treatment groups: Clothianidin 20-ppb (orange), clothianidin 5-ppb (green), control (blue) across 3 experiments. The gray area shows the treatment period. (**A**) AZ 2017; (**B**) AZ 2018; (**C**) MS 2018.

The effects of treatment and experiment were measured with respect to internal hive temperature and temperature amplitude for two periods: (1) from 1 Sept. to 31 Oct., to capture effects immediately after treatment during the active season, and (2) from 1 Nov. to 31 Dec., to capture effects as ambient conditions changed. Equipment failed from 12 to 25 Sept. in Mississippi so those dates were removed from the analysis. Treatment had no significant effect on average internal hive temperature (Fig. 6) or temperature variability (Fig. 7) but experiment did (Supplementary Tables S20-S27 online). Experiment as a factor had a significant effect on average hive temperature and temperature amplitude from 1 Sept. to 31 Oct., but only amplitude was affected in the 1 Nov. to 31 Dec. dataset.

### HIve CO_2_ concentration

**Figure 8:**
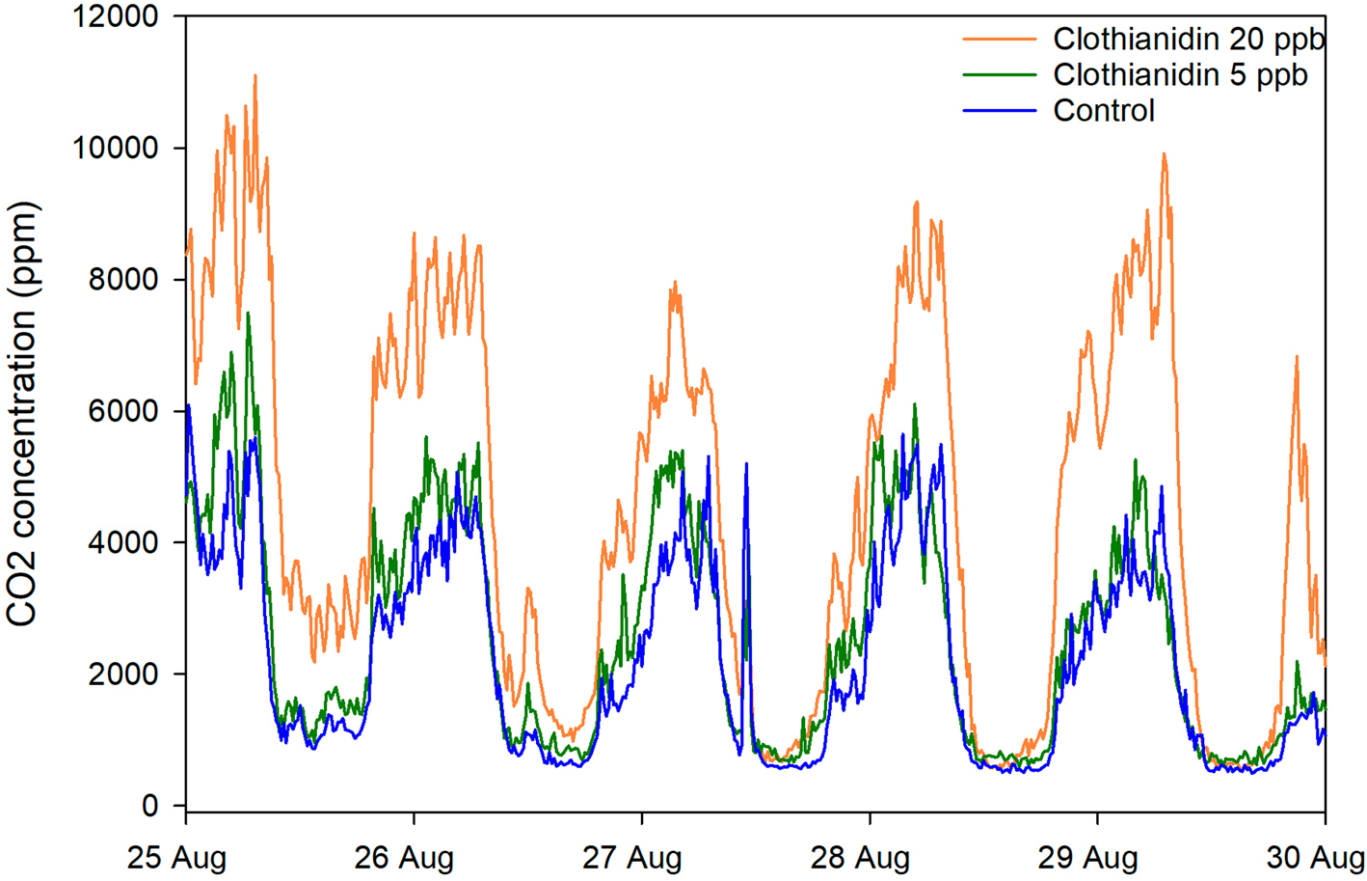
Average CO_2_ concentrations per treatment group for 5 days immediately after the end of treatment for each of 3 groups: Clothianidin 20-ppb (orange), clothianidin 5-ppb (green), and control (blue) for the AZ 2018 experiment.

**Figure 9:**
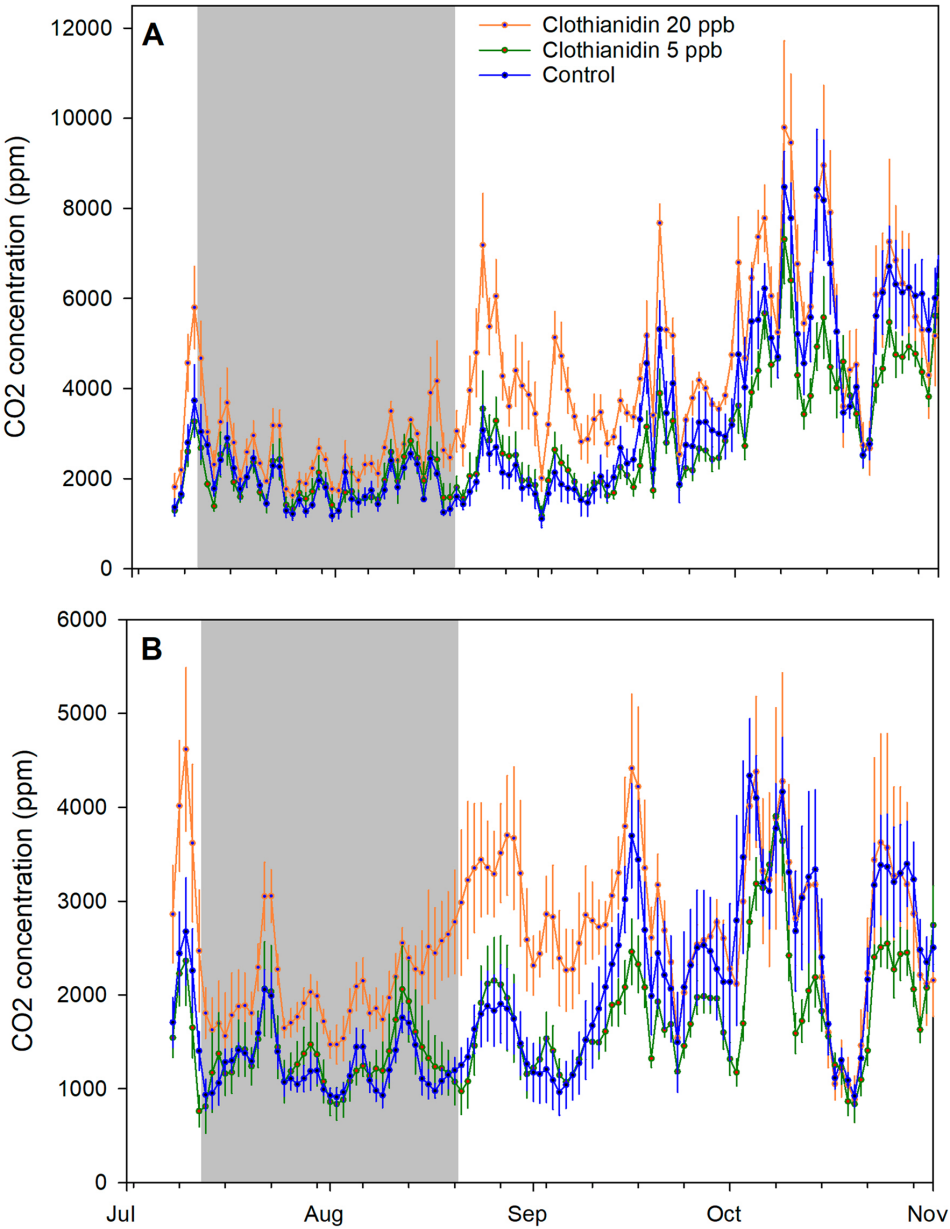
Running average CO_2_ concentrations, and daily amplitudes of sine curves fit to within-day CO_2_ concentration changes per day (see text for details), for each of 3 treatment groups: Clothianidin 20-ppb (orange), clothianidin 5-ppb (green), and control (blue) for the AZ 2018 experiment. The gray area shows the treatment period. (**A**) 24 h running average; (**B**) daily amplitudes.

Pre-treatment log CO_2_ average concentrations were significantly different among treatment groups (*p* = 0.0283) while pre-treatment concentration amplitudes were not (*p* = 0.068). Colonies had been ranked by adult bee mass and randomly assigned to groups, so the reason for these differences, which were detected after the experiments, was unclear. As with other analyses in this study, average pre-treatment values were used as covariates in the analysis of post-treatment data to control for pre-existing differences. Treatment had a significant effect (*p* = 0.0003) on average CO_2_ concentrations within the hive for at least the first two months after the end of the treatment period, from 1 Sept. to 31 Oct. (Figs. 8, 9, Supplementary Tables S28 and S29 online). Pairwise contrasts indicated that hives in the clothianidin 20-ppb treatment group had significantly higher CO_2_ concentration, 5002 ± 140 ppm, than either the clothianidin 5-ppb group, 3386 ± 91 ppm, (*p* = 0.0002) or the control group, 4109 ± 136 ppm (*p* = 0.0140). Treatment also affected CO_2_ concentration variability (amplitude) (*p* = 0.0041). Daily amplitudes within the hives ranged across treatment groups from 1933 to 2441 ppm, whereas amplitudes of ambient CO_2_ averaged 49 across the same time period.

### *Varroa* density

*Varroa* mite fall per hive was not affected by treatment group either in the AZ 2017 (*p* = 0.48) or AZ 2018 (*p* = 0.82) experiments (Table 3, Supplementary Table S30 online).

**Table 3:** Mite infestations per experiment. Mite levels in the two Arizona experiments were calculated as the number of mites fallen per colony per day; mite levels in the MS 2018 experiment were calculated as number of mites per 100 bees from samples of 300 bees.

Treatment	AZ 2017		AZ 2018			MS 2018
	July	October	July	October	November	May
Clothianidin 20-ppb	2.7 ± 0.9	7.8 ± 4.2	3.9 ± 1.3	18.7 ± 7.1	17.5 ± 6.7	8.9 ± 8.1
Clothianidin 5-ppb	2.0 ± 0.8	19.8 ± 11.6	3.4 ± 1.7	16.9 ± 6.7	37.1 ± 12.9	3.7 ± 1.1
Control	1.5 ± 0.7	15.3 ± 10.0	6.6 ± 2.3	14.3 ± 4.1	38.0 ± 18.7	5.6 ± 1.9

### Pesticide residue analyses

Residues in honey other than clothianidin were limited to thymol and trace amounts of 2,4-dimethylphenyl formamide (2,4-DMPF) in one sample (Table 4). Wax samples had many compounds but the residue concentrations were very low compared to acute contact LD_50_ (Supplementary Table S31 online).

**Table 4:** Concentrations of clothianidin and thymol in honey and syrup samples across treatment groups for the two experiments in Arizona. Values are parts per billion.

Year	Treatment group	Matrix	Thymol	Clothianidin			
				July	August	November	February
2017	Cloth_20	Honey	15	0	153	107	103
	Cloth_05	Honey	23	0	42	34	18
	Control	Honey	89*	0	0	0	0
	Cloth_20	Syrup		46			
	Cloth_5	Syrup		12			
2018	Cloth_20	Honey	55	0	22	12	7
	Cloth_05	Honey	–	0	trace	trace	0
	Control	Honey	–	0	0	0	0
	Cloth_20	Syrup		33			
	Cloth_5	Syrup		12			

*Trace amounts of 2,4-DMPF were also detected.

### Landscape analysis of Mississippi apiary

Analysis of the Poplarville, MS, landscape using CropScape yielded the usage patterns within about 1.8 km of the apiary (Supplementary Table S32 online), or an area of about 1018 ha. Agriculture (soybeans, corn, cotton, non-alfalfa hay, sweet potatoes and grass seed production) covered about 0.3% of the area, or about 3.1 ha. Uncultivated land, including forest, shrubland, wetland and pasture, covered 72.5% of the area. The remaining area, 27.2% of the total, was classified as “developed” to varying degrees, or open water.

## Discussion

The objective of this study was to compare honey bee colony size, growth and behavior among groups subjected to sublethal concentrations of clothianidin over three trials, including two conducted in consecutive years at the same site in Arizona and another conducted at a site in Mississippi. Response variables in all three trials included discrete measurements, i.e. adult bee mass, brood levels, and food resources, as well as continuous measurements, i.e. hive weight and internal hive temperature. Both treatment group and experiment (year and location) were used as factors in the analyses of those data. Two response variables, newly-emerged bee (NEB) weights and hive CO_2_ concentration, were collected only at the Arizona site. Clothianidin exposure had a significant impact on adult bee mass across all three rials at the first sampling occasion post-treatment, and colonies fed 20-ppb clothianidin had, on average, about 0.5 kg fewer adult bees, or about 21% less, than did those in the control group. Fewer adult bees would result in lower food consumption, and likely reduced foraging. However, clothianidin exposure did not have a significant effect on brood levels, food resources, daily hive weight change, or on average hive temperature or temperature variability.

Part of the difficulty in detecting treatment effects across all three trials may have been due to environmental variability between years and sites. The Arizona site was higher (about 1100 m) than the Mississippi site (95 m). Only 286 mm of precipitation fell during AZ 2017, reducing bee forage that year, while almost twice as much fell during AZ 2018 and almost 5 times as much fell during MS 2018. Between the pre-treatment hive inspection and the first post-treatment inspection, colonies in MS 2018 gained on average about 12.2 kg in food resources and colonies in AZ 2018 gained about 11.6 kg, compared to colonies in AZ 2017 which gained only about 6.2 kg. Hives in AZ 2017 also lost over 180 g per day on average for the first two months post-treatment, while hives in AZ 2018 lost only 6 g per day on average and hives in MS 2018 gained 6 g per day. Poor forage in AZ 2017 likely affected residue concentrations in the stored honey. Clothianidin was stable in honey for several months after the end of treatment, as has been found with imidacloprid^27^, and clothianidin residues in stored honey were much higher in AZ 2017 than the following year, suggesting less dilution from alternative nectar sources and a higher dose per bee.

Environmental factors also significantly affected internal hive temperature. Average daily hive temperatures were significantly lower for two months post-treatment in AZ 2017 compared to the other trials. Daily temperature amplitudes, reflecting variability, were significantly lower for colonies in Mississippi than colonies in Arizona for up to four months post-treatment, indicating more stable temperatures in the Mississippi hives. Treatment had no measurable effect on hive temperature.

In an effort to reduce variability and exploit the data further for treatment effects, further analyses focused on the Arizona trials. In Arizona, clothianidin exposure significantly affected adult bee mass, although post hoc comparisons were not significant. Lower adult bee numbers may have played a role in the hive weight data in Arizona; colonies fed 20-ppb clothianidin lost significantly more weight, 127 g per day on average, over two months post-treatment, than colonies fed 5-ppb clothianidin, 90 g per day. Lower daily hive weight loss in the 5-ppb group indicated either better foraging, reduced consumption or some combination of the two, compared to the 20-ppb group. Neither brood levels nor food resource levels were different between the two Arizona trials.

The dry weight of NEBs, measured only in Arizona, was about 6–7% lower for the 5-ppb treatment group than the control group, and was consistent across two years. The lower weight may have been due to lower consumption as larvae (NEBs were collected before they could feed as adults) or other factors. Low adult body weight has been associated with nutritional deficiencies^43^. Similar results have been observed in a study involving foragers exposed as larvae to clothianidin at about the same concentrations^4^. One possibility, although not investigated here, is that some treatment effects may be due to hormesis, defined as a change in the shape of the dose–response curve at low, sublethal concentrations of toxic compounds^46,47^ and thought to be rooted in oxidative stress protective mechanisms^48^. Effects observed at lower concentrations may be different to those observed at higher concentrations. For example, while high concentrations of nicotine, which attacks the same acetylcholine receptors as clothianidin, reduce honey bee colony survival, low concentrations have been found to improve honey bee short term memory and survival^49,50^.

Hive CO_2_ concentration was monitored in AZ 2018 as an indicator of colony-level organization. Hive CO_2_ concentrations are generally higher and more variable compared to ambient conditions; in Arizona hive CO_2_ concentrations averaged > 3700 ppm across all hives while ambient values averaged 409 ppm, and concentrations varied on average > 1900 ppm (often > 5000 ppm) while ambient values varied about 49 ppm. Managing CO_2_ levels is an important colony function; elevated CO_2_ concentrations can affect honey bee memory, ovary development and gene expression^51,52^. Treatment affected hive CO_2_ concentrations; average concentrations in the 20-ppb group were about 22% higher compared to the control group for two months after treatment. Since hive concentration depends on CO_2_ production as well as ventilation, colonies in the 20-ppb group either produced more CO_2_, or did not ventilate the hive the same way, or both. Daily variability in CO_2_ concentration was also higher in the 20-ppb group, at least compared to the 5-ppb group, suggesting that colonies in the 20-ppb group may have also ventilated more. Significant differences among groups were also observed before treatment, and although pre-treatment values were used as covariates to control for those differences, why those differences existed was not clear.

Environmental variability among experiments may have made effects due to pesticide exposure harder to detect. Results from MS 2018 were consistent with studies conducted in the southern half of the U.S. that have shown higher growth rates, better thermoregulation and lower pathogen loads for bee colonies kept in landscapes with some agriculture compared to those kept in other landscapes (urban, peri-urban, or completely unmanaged, depending on the study)^53–55^. The amount of agriculture within a 1.8 km radius of the Mississippi site was not high, but the density and diversity of honey bee forage was clearly higher than in southern Arizona. In contrast, other studies conducted in northern U.S. have found that honey bee colonies exposed to commercial agriculture reported higher levels of detoxification enzymes and poorer thermoregulation compared to colonies kept on non-agricultural Conservation Reserve Program land^56,57^.

These results indicate that, considering all three experiments, exposure to a 20-ppb concentration of clothianidin reduced average adult bee mass per colony by about 21% but had no significant effects on brood levels, food resources or thermoregulation. Considering only the Arizona trials, significant effects were observed with respect to adult bee mass, daily hive weight change, NEB weights and CO_2_ concentration. Some results were more difficult to interpret: daily hive weight loss in the fall was about 41% higher in colonies exposed to 20-ppb clothianidin than those exposed to 5-pbb clothianidin, but neither group was different from the control, and NEB weights were affected by a 5-ppb clothianidin exposure but not by a 20-ppb exposure. Those results suggest that the dose–response curve may change at low concentrations. Continuous data gathered over several months, such as hive weight and internal temperature, were easier to harmonize for analysis than discrete data that depended on similar hive assessment dates, and were statistically powerful due to frequent sampling. It is hoped that gathering more different kinds of data, particularly on the colony level, might provide further clues in understanding the relationships among bees, stressors and landscapes.

## Conclusions

1. Exposure of bee colonies to 20-ppb in sugar syrup clothianidin reduced average adult bee mass per colony by 21% and increased within-hive CO_2_ concentration by about 22%, compared to control colonies;
2. Exposure of colonies to a 5-ppb clothianidin concentration reduced NEB dry weight, but exposure to 20-ppb had no measurable effect;
3. Hive temperature profiles were not significantly affected by clothianidin exposure but did differ significantly among replicate trials, likely due to environmental differences; and
4. Clothianidin was very stable in honey within the hive over at least 6 months.

## Materials and methods

### Syrup preparation

Control (0 ppb clothianidin) sucrose solution was mixed at 1:1 w:w (e.g. 500 g sucrose:500 mL distilled water). Sucrose was added to distilled water in a 5-gallon bucket and mixed using an electric drill with a mortar mixing attachment until sugar was completely dissolved. Sucrose solution for solutions with clothianidin (PESTANAL, CAS# 210880-92-5) was mixed in the same manner but 50 mL was withheld (thus “short”) to allow for the added volume of respective clothianidin spikes. 500 g of sugar is dissolved in 450 mL of distilled water to allow for the addition of a 50 mL spike to achieve 1 kg of treatment solution. 950 g of “short” sugar solution was transferred to a Nalgene bottle, then the spike added to each individual bottle. A 10 ppm clothianidin stock solution was made by dissolving 1.0 mg of clothianidin, in 100 mL of distilled water, using a mixing bar but without heat. To avoid problems with static electricity, the clothianidin was weighed into a small, nonreactive plastic receptacles and those receptacles were placed in the solution, the solution stirred, and the receptacles removed after confirming the clothianidin had dissolved. For the 5-ppb solution: 0.5 mL of the stock solution was mixed into 49.5 mL of distilled water to achieve 50 mL of spike solution, which was then added to 950 g of the short sucrose solution to achieve 1 kg of 5-ppb clothianidin syrup. For the 20-ppb solution (only in 2nd experiment) 2.0 mL of stock solution was mixed into 48.0 mL of distilled water, and that solution added to 950 g of the short solution to achieve 1 kg of 20-ppb clothianidin syrup.

### AZ 2017 experiment

On 20 April, 2017, 24 bee colonies were established from packages of Italian honey bees (*A. mellifera ligustica*) (C.F. Koehnen & Sons, Inc., Glenn, CA 95,943) of approximately 1 kg honey bees in painted, 10-frame, wooden Langstroth boxes (43.7 l capacity) (Mann Lake Ltd., Hackensack, MN) with migratory wooden lids. At establishment, each colony was given 4 full or partial frames of capped honey, 2 frames of drawn but empty comb, 2 frames of partially drawn with some capped honey, 3 frames of foundation and a 1-frame feeder. Queens were marked, and during the course of the studies any queen replacements, such as for supersedure queens, was done with queens from the same breeder. Hives were placed on stainless steel electronic scales (Tekfa model B-2418 and Avery Weigh-Tronix model BSAO1824-200) (max. capacity: 100 kg, precision: ± 20 g; operating temperature: − 30 °C to 70 °C) and linked to 16-bit dataloggers (Hobo UX120-006 M External Channel datalogger, Onset Computer Corporation, Bourne, MA) with weight recorded every 5 min. The scales were powered by deep-cycle batteries connected to solar panels. The system had an overall precision of approximately ± 20 g. Hives were arranged in a circular pattern around a central box that contained the batteries and electronic gear. Hives within such a group were 0.5- 1 m apart and groups were > 3 m apart. During the course of the experiments the power systems had occasional malfunctions, resulting in short periods of missing data for some hives.

Colonies were all fed 2 kg sugar syrup (1:1 w:w) and 250 g pollen patty, made at a ratio of 1: 1: 1 corbicular pollen (Great Lakes Bee Co.): granulated sugar: drivert sugar (dry fondant sugar with approximately 8% invert sugar) (Domino Foods, Yonkers, NY). On 10 July a temperature sensor (iButton Thermochron, precision ± 0.06 °C) enclosed in plastic tissue embedding cassettes (Thermo Fisher Scientific, Waltham, MA) was stapled to the center of the top bar on the 5th frame in the bottom box of each hive and set to record every 15 min. The same day, pieces of slick paperboard coated with petroleum jelly and covered with mesh screens were inserted onto the hive floor to monitor *Varroa* mite fall within the hive^58^. The boards were removed 2 days later, and the number of mites counted on each board. Infestation levels of *Varroa* were again monitored during and post-treatment. Colonies were treated with amitraz (Apivar, Arysta LifeScience America Inc., New York, NY) on 19 October.

Hives were assessed on 12 July, and approximately every 5–6 weeks thereafter until November, the again 13 February 2020 and finally on 29 March using a published protocol^21,28^. Briefly, the hive was opened after the application of smoke, and each frame was lifted out sequentially, gently shaken to dislodge adult bees, photographed using a 16.3 megapixel digital camera (Canon Rebel SL1, Canon USA, Inc., Melville, NY), weighed on a portable scale (model EC15, OHaus Corp., Parsippany, NJ), and replaced in the hive. Frame photographs were analyzed later in the laboratory (see below). During this first assessment (but not subsequent assessments), all hive components (i.e. lid, inner cover, box, bottom board, frames, entrance reducer, internal feeder) were also shaken free of bees and weighed to yield an initial mass of all hive components. At the initial inspection, 3–5 g of wax and honey were collected from each hive into 50 ml centrifuge tubes and stored at − 80 °C; samples collected in September, prior to treatment, were pooled and subjected to a full panel analysis for residues of pesticides and fungicides, from all major classes, by the Laboratory Approval and Testing Division, Agricultural Marketing Service, USDA. Wax samples were collected only at the initial assessment in order to establish a baseline exposure—the lack of agriculture or landscaping within foraging distance excluded the possibility of further exposure. Honey samples from later assessments were pooled within treatment group and subjected only to neonicotinoid residue analysis.

Newly-emerged bees (NEBs) were sampled by pressing an 8 cm × 8 cm × 2 cm cage of wire mesh into a section of capped brood, then returning the following day to collect NEBs that had emerged within the cage over the previous 24 h. The NEBs were then placed in a 50 mL centrifuge tube, frozen on dry ice, and stored at − 80 °C. At the laboratory, 5 bees per hive per assessment date were placed in Eppendorf tubes, weighed, dried for 72 h at 60 °C, then re-weighed to determine average wet and dry weight per bee. NEBs were collected on 12 July and 24 August 2017 (brood levels were too low in October 2017 for sampling).

After the first assessment, hives were ranked with respect to adult bee mass and then randomly assigned to treatment group, ensuring that the average bee masses per group were approximately equal and after eliminating assignments that resulted in excessive spatial clumping of the colonies. Just prior to treatment all broodless frames containing honey and/or pollen were replaced with frames of empty drawn comb collected earlier from the same apiary. Colonies were then fed 2–3 kg syrup twice per week from 14 July to 21 August, with clothianidin concentrations depending on their treatment group. Syrup consumption per colony was recorded. Hives were assessed approximately every 5–6 weeks thereafter until November, and again in February and March. At each of those subsequent assessments, the same protocol was followed but only the frames, hive lid and inner cover were weighed. The hive lid and inner cover weights were compared to previous values and used to correct for moisture content changes in the hive components and improve estimates of adult bee mass. Food resources in the colonies were very low by mid-November so all colonies were provided with an additional 2 kg sugar syrup at that time.

### AZ 2018 experiment

The 2017–2018 experiment was conducted in the same manner, with the same or similar equipment and using the same bee suppliers. *Varroa* mite fall onto adhesive boards was monitored 6–9 July, and hives were assessed and sampled on 5 July in the same manner as before. NEBS were sampled on 6 July, 23 August and 4 October, 2018. CO_2_ probes (Vaisala Inc., Helsinki, Finland), calibrated for 0–20% concentrations, were installed in five hives in each treatment group and set to record CO_2_ concentration every 5 min. Colonies were fed 3 kg sugar syrup twice per week from 12 July to 20 August with the same pesticide concentrations as the previous year and assessed as before, with the experiment ending in February. *Varroa* infestation levels were monitored at the end of August and again at the beginning of November. Colonies were treated with amitraz (Apivar) on 19 October. Unlike the previous year, colonies were found to have sufficient resources to last to spring and so they were not fed any additional syrup after the treatment period.

### MS 2018 experiment

Full bee colonies, each comprised of two “deep” boxes as described above, were obtained from a local bee supplier (Gunter Apiaries, Lumberton MS) as nucleus colonies the previous year. Queens were bred locally and subspecies was unspecified. ^Colonies were placed on hive scales (Tekfa model B-2418) on 16 May 2018.^ Colonies were assessed, using the methods described above, on 11 July 2018 and temperature sensors (iButtons) were installed on 12 July 2018. Frames of honey were removed on 18 July and colonies were randomly placed in treatment groups. Treatment feeding commenced 24 July, lasting 31 August, using the same concentrations and amounts as described above. Colonies were not fed pollen patty because sufficient pollen was available. Colonies were assessed again 18 September 2018 and finally on 27 March 2019. Samples of 300 bees were collected on 7 May 2018, washed in 70% ethanol and the *Varroa* mites counted. Colonies were treated for *Varroa* (Checkmite, Mann Lake Ltd) on 28 June 2018. The apiary site was assessed using the National Agricultural Statistical Service (NASS) Cropscape web site (https://nassgeodata.gmu.edu/ CropScape) to obtain acreage estimates for all land use categories within an approximately 1.8 km radius of the apiary. Bees can forage beyond that distance; the radius was chosen to provide a sufficient area (> 1000 ha) to be representative of the forage available to the bees.

### Data analysis

The total weight of the adult bee population was calculated by subtracting the combined weights of hive components (i.e. lid, inner cover, box, bottom board, frames, entrance reducer, internal feeder) obtained at the start of the experiment (model EC15, OHaus) from the total hive weight recorded the midnight prior to the inspection. The area of sealed brood per frame was estimated from the photographs using ImageJ version 1.47 software (W. Rasband, National Institutes of Health, USA) or CombCount^59^; this method has been described elsewhere^20,28^. Food resources in the colonies were calculated as the total frame weight, less (1) the mass of the brood (calculated at 0.77 g/cm^2^) and (2) the mass of an empty frame of drawn comb (555 g)^28^.

Honey bee colony survivorship was analyzed using Proc LifeReg (SAS Inc. 2002). Survivorship curves were generated for each treatment group within each experiment. Treatments compared using ANOVA (α = 0.05) (Proc Glimmix, SAS Inc. 2002), with experiment as a random factor, with respect to three parameters: (1) the 30th percentile; (2) the 50th percentile; and 3) a shape variable calculated by subtracting the 40th from the 30th percentile.

Daily hive weight change was calculated as the difference between the weight at midnight of a given day to the weight 23 h 55 min later. Continuous temperature data were divided into daily average data and within-day detrended data. Detrended data were obtained as the difference between the 24 h running average and the raw data. Sine curves were fit to 3-day subsamples of the detrended data, taken sequentially by day^28^. Curve amplitudes, representing estimates of daily variability, were reduced to a data point every 3 days, to ensure no overlap between data subsamples, for repeated measures MANOVA analyses. CO_2_ concentration data were treated in the same fashion.

Adult bee masses , brood surface area, and total food resources for the 1st sampling occasion after treatment were analyzed across all three experiments using ANOVA (Proc Glimmix, SAS Inc. 2002). Further analyses were conducted among the Arizona experiments across multiple sampling occasions using repeated-measure MANOVA (Proc Glimmix, SAS Inc. 2002). A similar approach was taken with newly-emerged bee weights, i.e. initial analysis across AZ 2017 and AZ 2018 using ANOVA for a single sampling occasion, then for AZ 2018 using MANOVA across two sampling occasions. Daily hive weight change, internal hive temperature average and variability (i.e. amplitudes of fit sine curves) and CO_2_ concentration average and variability were used as response variables in repeated-measures MANOVA with treatment, sampling date, experiment and day, and all 2-way interactions, as fixed effects and with pre-treatment values as a covariate to control for pre-existing differences. Proc Univariate was used with all response variables to inspect the data for normality. Log transformations were conducted where necessary to improve normality. Analyses of hive weight and temperature were limited to approximately 3 months after the end of treatment to focus on the active season, and consisted of omnibus tests that initially included all three field experiments followed by analyses within each experiment. The reason for this is that effects that are significant in one trial might not be so in another, or might be significant but in a contrary fashion. CO_2_ concentration data were only collected in AZ 2018.

NEB data were analyzed with Treatment, Sampling date and their interaction, with the July values as a covariate. *Varroa* fall were analyzed within each Arizona experiment, with the pre-treatment values used as covariates where applicable. *Varroa* alcohol wash data for MS 2018 were analyzed separately.

Rainfall, and ambient temperature and CO_2_ data, were obtained for the Arizona site: AmeriFlux US-SRM Santa Rita Mesquite, https://doi.org/10.17190/AMF/1246104; and temperature and rainfall data for Mississippi: National Environmental Satellite, Data, and Information Service, National Oceanic and Atmospheric Administration, Poplarville Experimental Station, MS US USC00227128.

## Supplementary Information

Supplementary Information.

Supplementary Tables.

## Data Availability

All raw data on hive assessment, temperature and weight are available in the Supplementary File online.
