# Supplementary material for: Sublethal concentrations of clothianidin affect honey bee colony growth and hive CO2 concentration
Source: Sci Rep. 2021 Feb 23;11:4364. doi: 10.1038/s41598-021-83958-8 (PMC7902615; doi:10.1038/s41598-021-83958-8)
Supplement: Supplementary file 2 — Supplementary Tables. [file 41598_2021_83958_MOESM2_ESM.pdf]

Sublethal concentrations of clothianidin affect honey bee colony growth and hive CO_2 concentration

William G. Meikle[1*], John J. Adamczyk[2], Milagra Weiss[1], Janie Ross[2], Chris Werle[2], Eli Beren[1]

[1]Carl Hayden Bee Research Center, USDA-ARS, 2000 E. Allen Rd, Tucson, AZ 85719 USA
[2]Southern Horticultural Laboratory, USDA-ARS, P. O. Box 287, Poplarville MS 39470 USA

*Corresponding author
E-mail: william.meikle@usda.gov

S1 Table. MANOVA results for the effects of syrup treatment, i.e. clothianidin 20 ppb, clothianidin 5 ppb, and control (blank) across 3 experiments, i.e. AZ 2017, AZ 2018, and POPL 2018, the first sampling occasion after the end of treatment, on average adult bee mass (kg) per colony. Pre-treatment adult bee mass was used as a covariate to control for pre-existing differences among colonies.

Factor	Num DF	Den DF	F Value	Pr > F
Treatment	2	40	3.88	0.0289
Experiment	2	40	4.18	0.0224
Treat* Experiment	4	40	1.89	0.1313
Pre-treat adult bee mass	1	40	5.27	0.0270

S2 Table. Post hoc contrasts among treatment groups for S1 Table above. “Cloth_20” refers to treatment group exposed to clothianidin at 20 ppb in sugar syrup, “Cloth_05” refers to group exposed to clothianidin at 5 ppb, and “Control” refers to the untreated control group.

Factor	Contrast	Estimate	Standard Error	DF	t Value	Adj P
Treatment	Cloth_20 vs Cloth_05	-0.07915	0.2886	40	-0.27	1.0000
	Cloth_20 vs Control	-0.6901	0.2754	40	-2.51	0.0492
	Cloth_05 vs Control	-0.6110	0.2758	40	-2.21	0.0976
Experiment	MS 2018 vs AZ 2017	-0.5058	0.4750	40	-1.06	0.8801
	MS 2018 vs AZ 2018	-0.9936	0.4086	40	-2.43	0.0588
	AZ 2017 vs AZ 2018	-0.4878	0.2776	40	-1.76	0.2596

S3 Table. MANOVA results for the effects of syrup treatment, i.e. clothianidin 20 ppb, clothianidin 5 ppb, and control (blank) across 2 experiments, i.e. AZ 2017 and AZ 2018, and 4 sampling occasions on average adult bee mass (kg) per colony. Hive number was a random factor and pre-treatment adult bee mass was used as a covariate to control for pre-existing differences among colonies.

Effect	Num DF	Den DF	F Value	Pr > F
Treatment	2	32.22	3.40	**0.0456**
Experiment	1	31.87	0.45	0.5053
Sampling occasion	3	78.54	106.24	<0.0001
Treat*SO	6	78.68	1.84	0.1025
Treat* Experiment	2	31.46	0.57	0.5712
SO* Experiment	3	78.52	10.39	<0.0001
Pre-treat adult bee mass	1	30.91	10.88	0.0024

S4 Table. Post hoc contrasts among treatment groups for S3 Table above. “Cloth_20” refers to treatment group exposed to clothianidin at 20 ppb in sugar syrup, “Cloth_05” refers to group exposed to clothianidin at 5 ppb, and “Control” refers to the untreated control group.

Contrast	Estimate	Standard Error	DF	t Value	Adj P
Cloth_20 vs Cloth_05	-0.4337	0.2176	33.12	-1.99	0.1642
Cloth_20 vs Control	-0.5334	0.2161	32.47	-2.47	0.0571
Cloth_05 vs Control	-0.09972	0.2137	31.16	-0.47	1.0000

S5 Table. MANOVA results for the effects of syrup treatment, i.e. clothianidin 20 ppb, clothianidin 5 ppb, and control (blank) across 3 experiments, i.e. AZ 2017, AZ 2018, and POPL 2018, the first sampling occasion after the end of treatment, on average surface area of capped brood per colony (cm^2). Pre-treatment adult bee mass was used as a covariate to control for pre-existing differences among colonies.

Factor	Num DF	Den DF	F Value	Pr > F
Treatment	2	40	0.68	0.5131
Experiment	2	40	1.34	0.2729
Treat* Experiment	4	40	0.54	0.7098
Pre-treat brood area	1	40	6.58	0.0142

S6 Table. MANOVA results for the effects of syrup treatment, i.e. clothianidin 20 ppb, clothianidin 5 ppb, and control (blank) across 2 experiments, i.e. AZ 2017 and AZ 2018 and 4 sampling occasions on capped brood surface area (cm^2) per colony. Hive number was a random factor and pre-treatment brood area was used as a covariate to control for pre-existing differences among colonies.

Effect	Num DF	Den DF	F Value	Pr > F
Treatment	2	24.87	0.61	0.5532
Experiment	1	25.21	2.59	0.1197
Sampling Occasion (SO)	3	70.70	58.22	<0.0001
Treat*Experiment	2	24.16	0.25	0.7798
Treat*SO	6	71.33	0.38	0.8925
SO*Experiment	3	70.53	17.20	<0.0001
Pre-treat brood area	1	25.64	5.96	0.0219

S7 Table. MANOVA results for the effects of syrup treatment, i.e. clothianidin 20 ppb, clothianidin 5 ppb, and control (blank) across 3 experiments, i.e. AZ 2017, AZ 2018, and POPL 2018, at the first sampling occasion after the end of treatment, on average food resources (kg) per colony.

Factor	Num DF	Den DF	F Value	Pr > F
Treatment	2	39	1.01	0.3724
Experiment	2	39	14.34	<0.0001
Treat* Experiment	4	39	0.57	0.6879
Pre-treat food resources	1	39	6.20	0.0172

S8 Table. Post hoc contrasts among treatment groups for S7 Table above.

Contrast	Estimate	Standard Error	DF	t Value	Adj P
MS 2018 vs AZ 2017	8.42	1807.52	39	4.66	0.0001
MS 2018 vs AZ 2018	2.70	1680.31	39	1.61	0.3466
AZ 2017 vs AZ 2018	-5.72	1283.49	39	-4.46	0.0002

S9 Table. MANOVA results for the effects of syrup treatment, i.e. clothianidin 20 ppb, clothianidin 5 ppb, and control (blank) across 2 experiments, i.e. AZ 2017 and AZ 2018 on the change in total food resources (g) per colony over 3 sampling occasions. Hive number was a random factor.

Effect	Num DF	Den DF	F Value	Pr > F
Treatment	2	87	0.84	0.4346
Experiment	1	87	31.76	<0.0001
Sampling Occasion (SO)	2	62.21	9.69	0.0002
Treat*Experiment	2	87	1.54	0.2195
Treat*SO	4	69.49	0.52	0.7214
SO*Experiment	2	62.21	7.99	0.0008
Pre-treat food resources	1	87	7.57	0.0072

S10 Table. Post hoc contrasts among treatment groups for S9 Table above. "2017" refers to the AZ 2017 experiment and "2018" refers to the AZ 2018 experiment.

Contrast								
Sampling occasion	Year	Sampling occasion	Year	Estimate	Standard Error	DF	t Value	Adj P
1	2017	1	2018	-5.52	1052.41	87	-5.25	<0.0001
2	2017	2	2018	-8.76	1647.58	87	-5.32	<0.0001
3	2017	3	2018	-10.66	1941.29	87	-5.49	<0.0001

S11 Table. MANOVA results for the effects of syrup treatment, i.e. clothianidin 20 ppb, clothianidin 5 ppb, and control (blank) on Newly Emerged Bee (NEB) dry weights (g) post treatment across 2 experiments, i.e. AZ 2017 and AZ 2018. Weight values were the averages of 10 bees per colony per sampling occasion. Hive number was a random factor and pre-treatment NEB dry weight was used as a covariate to control for pre-existing differences among colonies.

Effect	Num DF	Den DF	F Value	Pr > F
Treatment	2	24	3.65	0.0413
Experiment	1	24	1.40	0.2485
Treatment*Experiment	2	24	2.53	0.1008
Pre-treat NEB dry wt	1	24	9.77	0.0046

S12 Table. Post hoc contrasts among treatment groups for S11 Table above. "Cloth_20" refers to treatment group exposed to clothianidin at 20 ppb in sugar syrup, "Cloth_05" refers to group exposed to clothianidin at 5 ppb, and "Control" refers to the untreated control group.

Contrast	Estimate	Standard Error	DF	t Value	Adj P
Cloth_20 vs Cloth_05	0.000456	0.000563	24	0.81	1.0000
Cloth_20 vs Control	-0.00092	0.000539	24	-1.71	0.2988
Cloth_05 vs Control	-0.00138	0.000524	24	-2.63	0.0440

S13 Table. MANOVA results for the effects of syrup treatment, i.e. clothianidin 20 ppb, clothianidin 5 ppb, and control (blank) on Newly Emerged Bee dry weights (g) across 2 post-treatment sampling occasions for the AZ 2018 experiment. Weight values were the averages of 10 bees per colony per sampling occasion. Hive number was a random factor and pre-treatment NEB dry weight was used as a covariate to control for pre-existing differences among colonies.

Effect	Num DF	Den DF	F Value	Pr > F
Treatment	2	15.63	7.75	0.0046
Sampling occasion	1	25.75	0.02	0.9009
Treatment* SO	2	25.45	0.31	0.7391
Pre-treat NEB dry wt	1	11.46	12.67	0.0042

S14 Table. Post hoc contrasts among treatment groups for S13 Table above. “Cloth_20” refers to treatment group exposed to clothianidin at 20 ppb in sugar syrup, “Cloth_05” refers to group exposed to clothianidin at 5 ppb, and “Control” refers to the untreated control group.

Contrast	Estimate	Standard Error	DF	t Value	Adj P
Cloth_20 vs Cloth_05	0.001395	0.000479	14.91	2.91	0.0310
Cloth_20 vs Control	-0.00030	0.000465	15.78	-0.65	1.0000
Cloth_05 vs Control	-0.00170	0.000453	16.18	-3.75	0.0054

S15 Table. MANOVA results for the effects of syrup treatment, i.e. clothianidin 20-ppb, clothianidin 5-ppb, and control (blank) for the experiment conducted in MS 2018 on hive weight change (g) per colony from 1 Sept. to 31 Oct. Hive number was a random factor and pre-treatment adult bee mass was used as a covariate to control for pre-existing differences.

Effect	Num DF	Den DF	F Value	Pr > F
Treatment	2	218.9	0.12	0.8908
Experiment	2	220.9	32.14	<0.0001
Day	56	2206	22.64	<0.0001
Treatment*Day	112	2255	1.07	0.2944
Treatment*Experiment	4	225.1	2.68	0.0324
Day* Experiment	112	2255	23.21	<0.0001
Pre-treat adult bee mass	1	225.3	2.73	0.1001

S16 Table. Post hoc contrasts among experiments for S15 Table above.

Contrast	Estimate	Standard Error	DF	t Value	Adj P
MS 2018 vs AZ 2017	0.1164	0.02904	222.5	4.01	0.0003
MS 2018 vs AZ 2018	-0.03290	0.02501	221.8	-1.32	0.5689
AZ 2017 vs AZ 2018	-0.1493	0.01863	219.5	-8.01	<.0001

S17 Table. MANOVA results for the effects of syrup treatment, i.e. clothianidin 20 ppb, clothianidin 5 ppb, and control (blank) across the 2 experiments in Arizona, i.e. AZ 2017 and AZ 2018 on daily hive weight change (g) per colony from 1 Sept. to 31 Oct. Hive number was a random factor and pre-treatment adult bee mass was used as a covariate to control for pre-existing differences.

Effect	Num DF	Den DF	F Value	Pr > F
Treatment	2	392.3	3.54	0.0301
Experiment	1	393.5	117.60	<0.0001
Day	54	1496	36.07	<0.0001
Treatment*Day	108	1490	1.25	0.0470
Treatment*Experiment	2	396.5	2.59	0.0759
Day* Experiment	54	1496	29.72	<0.0001
Pre-treat adult bee mass	1	396.2	0.68	0.4093

S18 Table. Post hoc contrasts among treatment groups for S17 Table above. "Cloth_20" refers to treatment group exposed to clothianidin at 20 ppb in sugar syrup, "Cloth_05" refers to group exposed to clothianidin at 5 ppb, and "Control" refers to the untreated control group.

Contrast	Estimate	Standard Error	DF	t Value	Adj P
Cloth_20 vs Cloth_05	-0.03958	0.01489	392.3	-2.66	0.0245
Cloth_20 vs Control	-0.01889	0.01412	392.1	-1.34	0.5449
Cloth_05 vs Control	0.02069	0.01468	392.4	1.41	0.4785

S19 Table. MANOVA results for the effects of syrup treatment, i.e. clothianidin 20 ppb, clothianidin 5 ppb, and control (blank) across the 2 experiments in Arizona, i.e. AZ 2017 and AZ 2018 on daily hive weight change (g) per colony from 1 Dec. to 31 Jan. Hive number was a random factor and pre-treatment adult bee mass was used as a covariate to control for pre-existing differences.

Effect	Num DF	Den DF	F Value	Pr > F
Treatment	2	274.5	0.03	0.9711
Experiment	1	275.3	0.27	0.6023
Day	61	1598	89.73	<0.0001
Treatment*Day	122	1586	0.77	0.9653
Treatment*Experiment	2	280.3	1.24	0.2896
Day* Experiment	59	1598	90.57	<0.0001
Pre-treat adult bee mass	1	277.3	0.85	0.3584

S20 Table. MANOVA results for the effects of syrup treatment, i.e. clothianidin 20 ppb, clothianidin 5 ppb, and control (blank) across 3 experiments, i.e. AZ 2017, AZ 2018, and MS 2018, on average daily hive internal temperature (°C) for two months post-treatment (1 Sept. – 31 Oct.). Hive number was a random factor and pre-treatment adult bee mass was used as a covariate to control for pre-existing differences. Values every 3 days were used, to harmonize analysis with temperature amplitude analysis (see below).

Effect	Num DF	Den DF	F Value	Pr > F
Treatment	2	37.31	0.92	0.4084
Experiment	2	37.04	28.25	<0.0001
Day	15	566.4	36.79	<0.0001
Treatment* Day	30	583	1.15	0.2678
Treatment*Experiment	4	35.34	0.55	0.7020
Day * Experiment	30	583	23.11	<0.0001
Pre-treat average temperature	1	35.34	10.16	0.0030

S21 Table. Post hoc contrasts among experiments for S20 Table above.

Contrast	Estimate	Standard Error	DF	t Value	Adj P
MS 2018 vs AZ 2017	2.4226	0.4699	36.74	5.16	<0.0001
MS 2018 vs AZ 2018	-0.4584	0.4159	37.09	-1.10	0.8325
AZ 2017 vs AZ 2018	-2.8810	0.3895	37.18	-7.40	<0.0001

S22 Table. MANOVA results for the effects of syrup treatment, i.e. clothianidin 20 ppb, clothianidin 5 ppb, and control (blank) across 3 experiments, i.e. AZ 2017, AZ 2018, and MS 2018, on average daily hive internal temperature (°C) for two months post-treatment (1 Nov. – 31 Dec.). Hive number was a random factor and pre-treatment adult bee mass was used as a covariate to control for pre-existing differences. Values every 3 days were used, to harmonize analysis with temperature amplitude analysis (see below).

Effect	Num DF	Den DF	F Value	Pr > F
Treatment	2	37	0.37	0.6931
Experiment	2	37.35	0.70	0.5019
Day	19	729.7	10.04	<0.0001
Treatment* Day	38	730.7	0.44	0.9987
Treatment*Experiment	4	35.74	0.21	0.9310
Day * Experiment	38	730.7	20.73	<0.0001
Pre-treat average temperature	1	39.01	1.11	0.2996

S23 Table. Post hoc contrasts among experiments for S22 Table above.

Contrast	Estimate	Standard Error	DF	t Value	Adj P
MS 2018 vs AZ 2017	0.04164	0.04915	37.79	0.85	1.0000
MS 2018 vs AZ 2018	0.05050	0.04299	36.77	1.17	0.7428
AZ 2017 vs AZ 2018	0.008862	0.04076	37.64	0.22	1.0000

S24 Table. MANOVA results for the effects of syrup treatment, i.e. clothianidin 20 ppb, clothianidin 5 ppb, and control (blank) across 3 experiments, i.e. AZ 2017, AZ 2018, and MS 2018, on daily hive internal temperature amplitudes (°C) for two months post-treatment (1 Sept. – 31 Oct.). Hive number was a random factor and pre-treatment adult bee mass was used as a covariate to control for pre-existing differences. Amplitudes were calculated from 3-day datasets so values every 3 days were used.

Effect	Num DF	Den DF	F Value	Pr > F
Treatment	2	38.34	1.60	0.2159
Experiment	2	38.19	83.71	<0.0001
Day	15	525.6	6.97	<0.0001
Treatment* Day	30	557.8	1.07	0.3636
Treatment*Experiment	4	37.02	0.75	0.5664
Day * Experiment	30	557.8	6.48	<0.0001
Pre-treat adult bee mass	1	37.02	3.33	0.0760

S25 Table. Post hoc contrasts among experiments for S24 Table above.

Contrast	Estimate	Standard Error	DF	t Value	Adj P
MS 2018 vs AZ 2017	-0.7627	0.07460	38.2	-10.22	<0.0001
MS 2018 vs AZ 2018	0.1929	0.07000	38.33	2.76	0.0268
AZ 2017 vs AZ 2018	0.9556	0.07725	38.01	12.37	<0.0001

S26 Table. MANOVA results for the effects of syrup treatment, i.e. clothianidin 20 ppb, clothianidin 5 ppb, and control (blank) across 3 experiments, i.e. AZ 2017, AZ 2018, and MS 2018, on daily hive internal temperature amplitudes (°C) for two months post-treatment (1 Nov. – 31 Dec.). Hive number was a random factor and pre-treatment adult bee mass was used as a covariate to control for pre-existing differences. Amplitudes were calculated from 3-day datasets so values every 3 days were used.

Effect	Num DF	Den DF	F Value	Pr > F
Treatment	2	48.71	0.54	0.5864
Experiment	2	49.09	9.28	0.0004
Day	20	724.6	13.29	<0.0001
Treatment* Day	40	744	0.87	0.7081
Treatment*Experiment	4	47.35	0.51	0.7268
Day * Experiment	40	743.9	9.85	<0.0001
Pre-treat adult bee mass	1	48.44	0.80	0.3756

S27 Table. Post hoc contrasts among experiments for S26 Table above.

Contrast	Estimate	Standard Error	DF	t Value	Adj P
MS 2018 vs AZ 2017	-0.4802	0.1140	49.4	-4.21	0.0003
MS 2018 vs AZ 2018	-0.2739	0.1059	48.22	-2.59	0.0382
AZ 2017 vs AZ 2018	0.2063	0.1187	49.86	1.74	0.2651

S28 Table. MANOVA results for the effects of syrup treatment, i.e. clothianidin 20 ppb, clothianidin 5 ppb, and control (blank) for the AZ 2018 experiment on hive internal CO_2 average concentration (ppm) and CO_2 concentration amplitude (ppm) from 1 Sept to 31 Oct. Hive number was a random factor and pre-treatment adult bee mass was used as a covariate to control for pre-existing differences.

Response variable	Effect	Num DF	Den DF	F Value	Pr > F
CO_2 average concentration	Treatment	2	46.05	9.94	0.0003
	Day	61	810.3	25.37	<0.0001
	Pre-treat CO2 concentration	1	46.05	2.72	0.1058
CO_2 concentration amplitude	Treatment	2	31.48	6.58	0.0041
	Day	19	249.4	11.60	<0.0001
	Pre-treat CO2 concentration	1	31.48	3.44	0.0729

S29 Table. Post hoc contrasts among treatment groups for S28 Table above. "Cloth_20" refers to treatment group exposed to clothianidin at 20 ppb in sugar syrup, "Cloth_05" refers to group exposed to clothianidin at 5 ppb, and "Control" refers to the untreated control group.

Response variable	Contrast	Estimate	SE	DF	t Value	Adj P
CO_2 average concentration	Cloth_20 vs Cloth_05	1889.80	424.01	46.05	4.46	0.0002
	Cloth_20 vs Control	1199.59	403.32	46.05	2.97	0.0140
	Cloth_05 vs Control	-690.20	345.55	46.05	-2.00	0.1551
CO_2 concentration amplitude	Cloth_20 vs Cloth_05	1302.31	360.07	31.48	3.62	0.0031
	Cloth_20 vs Control	728.27	339.05	31.48	2.15	0.1185
	Cloth_05 vs Control	-574.03	292.43	31.48	-1.96	0.1756

S30 Table. MANOVA results for the effects of syrup treatment, i.e. clothianidin 20 ppb, clothianidin 5 ppb, and control (blank) on Varroa mite fall across 2 experiments, i.e. AZ 2017 and AZ 2018. Hive number was a random factor and pre-treatment Varroa mite fall was used as a covariate to control for pre-existing differences among colonies.

Effect	Num DF	Den DF	F Value	Pr > F
Treatment	2	27	1.20	0.3156
Experiment	1	27	0.00	0.9609
Treatment* Experiment	2	27	0.66	0.5234
Pre-treat Varroa count	1	27	16.45	0.0004

S31 Table. Pesticide concentrations in wax samples collected pre-treatment in the 2017-18 experiment in Arizona. Values are parts per billion. “LOD” means Limit of Detection; “DMPF” is dimethylphenyl formamide. Data on acute contact LD_{50} were obtained from the Pesticide Properties Database (https://sitem.herts.ac.uk/aeru/ppdb/en/atoz.htm) and converted from µg per bee to ppb assuming an average bee mass of 0.1g.

**Compound**	**LOD**	**Contact LD_{50} (ppb)**	**2017-18 Treatment group**			**2018-19**
			**Cloth_20**	**Cloth_5**	**Control**	**Composite**
2,4-DMPF	1.5	$7.50x10^5$	7	14	32	56
Boscalid	5	$>2.00x10^6$	5	-	-	-
Carbendazim	2	$>5.00 x10^5$	24	27	37	trace
Chlorthal-dimethyl	2	$>1.00 x10^6$	-	trace	-	-
Coumaphos oxon	1	$5.93x10^4$	1	1	1	trace
Cyprodinil	2	$>7.84 x10^6$	-	trace	-	-
Diuron	1	$>1.02 x10^6$	3	1	2	trace
Fenamidone	1	$>2.57 x10^5$	trace	-	-	-
Fenazaquin	1	$1.21 x10^4$	-	2	1	-
Fenpyroximate	3	$1.58 x10^5$	5	-	3	trace
Flumeturon	1	$>1.00 x10^6$	-	-	-	trace
Fluvalinate	25	$4.32 x10^4$	trace	trace	trace	trace
Hexythiazox	2	$>2.00 x10^6$	trace	trace	trace	trace
Pendimethalin	50	$1.00 x10^6$	-	-	trace	-
Piperonyl butoxide	6	NA	36	59	trace	trace
Propargite	2	$4.79 x10^5$	32	19	29	7
Thymol	2	NA	799	991	2190	1470
Trifluralin	10	$>1.00x10^6$	-	-	-	trace

S32 Table. Estimated surface area and percentage area for a circle with a radius of approximately 1.8 km (= approximately 1018 ha) of land around the Poplarville, MS, apiary in this study according to the Cropscape web site (see text for details).

Category	Area (ha)	Percentage
Corn	0.63	0.06
Cotton	0.51	0.05
Soybeans	1.50	0.14
Other Hay/Non Alfalfa	0.35	0.03
Sweet Potatoes	0.08	0.01
Sod/Grass Seed	0.08	0.01
Open Water	25.91	2.40
Developed/Open Space	138.50	12.85
Developed/Low Intensity	64.25	5.96
Developed/Medium Intensity	49.13	4.56
Developed/High Intensity	13.31	1.23
Barren	2.28	0.21
Deciduous Forest	2.09	0.19
Evergreen Forest	146.93	13.63
Mixed Forest	16.38	1.52
Shrubland	341.65	31.69
Grass/Pasture	129.41	12.00
Woody Wetlands	144.02	13.36
Herbaceous Wetlands	1.06	0.10
